# The ESX-5 Associated *eccB_5_*-*eccC_5_* Locus Is Essential for *Mycobacterium tuberculosis* Viability

**DOI:** 10.1371/journal.pone.0052059

**Published:** 2012-12-17

**Authors:** Mariagrazia Di Luca, Daria Bottai, Giovanna Batoni, Mickael Orgeur, Anna Aulicino, Claudio Counoupas, Mario Campa, Roland Brosch, Semih Esin

**Affiliations:** 1 Dipartimento di Patologia Sperimentale, Biotecnologie Mediche, Infettivologia ed Epidemiologia, University of Pisa, Pisa, Italy; 2 Dipartimento di Ricerca Traslazionale e delle Nuove Tecnologie in Medicina e Chirurgia, University of Pisa, Pisa, Italy; 3 Institut Pasteur, Unit for Integrated Mycobacterial Pathogenomics, Paris, France; University of Padova, Medical School, Italy

## Abstract

The recently described ESX-5 secretion system of *Mycobacterium tuberculosis* is one of the most important modulators of host-pathogen interactions due to its crucial impact on PPE protein secretion, cell wall stability and virulence. Although various components of the ESX-5 secretion machinery have been defined, other ESX-5 core components still remain to be characterized. In this study, we focused on EccB_5_ and EccC_5_, a transmembrane protein (EccB_5_) and a membrane-bound ATPase (EccC_5_), both predicted to be building blocks of the *M. tuberculosis* ESX-5 membrane-associated complex. *In vitro* expression studies demonstrated that EccB_5_ and EccC_5_ encoding genes constitute an operon. The expression of this operon is essential for *M. tuberculosis*, since the deletion of the *eccB_5_-eccC_5_* genomic segment at the ESX-5 locus is possible only after the integration of a second functional copy of *eccB_5_-eccC_5_* genes into the *M. tuberculosis* chromosome. The characterization of two *M. tuberculosis* conditional mutant strains (Mtb*_Pptr_eccB_5_* and Mtb*_Pptr_eccC_5_)*, in which the *eccB_5_-eccC_5_* operon or the *eccC_5_* gene, respectively, were expressed under the control of an anhydrotetracycline-repressible promoter, confirmed that the repression of *eccB_5_-eccC_5_* genes is detrimental for growth of *M. tuberculosis* both *in vitro* and in THP-1 human macrophage cell line. Moreover, analysis of the secretome of Mtb*_Pptr_eccB_5_-eccC_5_* and Mtb*_Pptr_eccC_5_* strains revealed that both EccB_5_ and EccC_5_ are required for secretion of ESX-5 specific substrates, thus confirming that they are indeed components of the ESX-5 secretion machinery. Taken together these findings demonstrate the importance of an intact and functional ESX-5 system for viability of *M. tuberculosis*, thus opening new interesting options for alternative antimycobacterial control strategies.

## Introduction

Throughout evolution, numerous bacterial pathogens have acquired specialized protein secretion pathways to deliver effector proteins to host cells. These pathways, which are distinct from the ubiquitous Sec pathway, are critical for mediating interactions during infection and for allowing pathogen survival in the hostile environment of the host. The recently described mycobacterial type VII secretion systems are specialized secretion systems of small, highly immunogenic proteins lacking a classical N-terminal signal sequence, belonging to the Esx or WXG-100 family [1], [2], which are distantly related to protein transport systems of Gram-positive bacteria [3]. The genome of *Mycobacterium tuberculosis*, the causative agent of human tuberculosis, encodes five type VII secretion systems (ESX-1 – ESX-5), the genes of which are arranged in highly conserved clusters [4]–[6]. Each ESX cluster typically carries a pair of *esx* genes flanked by *esx conserved components* (*ecc*) genes coding for predicted core components of the ESX secretion machineries responsible for the ATP-dependent transport of the corresponding ESX substrates outside the cell [5], [7]. ESX-5 is the most recently evolved ESX cluster and is only present in the group of slow-growing mycobacteria that includes all major pathogenic species [8]. In the fish pathogen *M. marinum*, ESX-5 modulates host-pathogen interactions [9], [10] and is responsible for secretion of several PPE and PE proteins, two of the most important classes of mycobacterial proteins involved in virulence/pathogenicity and implicated in immune evasion strategies used by pathogenic mycobacteria to survive in host tissues [11]–[15]. To date, PPE and PE proteins identified as being transported to the cell surface/secreted by the *M. marinum* ESX-5 are PE25-PPE41 [16], [17], LipY [18], and members of the PPE_MPTR and PE_PGRS subgroups [19], the most recent subclasses of PE and PPE proteins, genes of which were suggested to have evolved by duplication/insertion events from ancestral genes encoded at the ESX-5 locus [8]. By the characterization of several *M. tuberculosis* knock-out mutants for ESX-5 components, we recently demonstrated that ESX-5 plays a crucial role in host pathogen interaction also in *M. tuberculosis*. The *M. tuberculosis* ESX-5 system mediates PPE protein transport and secretion [20] with impact on T-cell immunogenicity of ESX-5- and non-ESX-5-encoded PPE and PE proteins. As such, ESX-5 is a major modulator of the host immune response [21] and a key virulence determinant of *M. tuberculosis*: inactivation of ESX-5 core components results in a strong attenuation of the corresponding mutant strains, which are unable to replicate both in immunodeficient and in immunocompetent mice [20], [21]. Various components of the ESX-5 secretion machinery, such as EccD_5_ (the predicted transmembrane channel) or EccA_5_ (a cytosolic ATPase belonging to the AAA+ family) have been characterized, and their impact on secretion of ESX-5 specific substrates has been investigated [20]. However, other putative building blocks of the ESX-5 secretion apparatus in *M. tuberculosis* still remain to be characterized. In this study, we focused on EccB_5_ and EccC_5_, a transmembrane protein and an ATP-binding protein belonging to the FtsK/SpoIIIE-like protein family, respectively [7], [22]. Encoded at the ESX-5 locus upstream of the *ppe25-pe19* cluster, EccB_5_ and EccC_5_ are both predicted to be components of the *M. tuberculosis* ESX-5-membrane-associated complex [7], [20], [23]. We demonstrated that *eccB_5_-eccC_5_* genes constitute an operon, expression of which is required for an efficient secretion of ESX-5 specific substrates. Moreover, by constructing/characterizing multi-copy gene variants and conditional mutants, in which the *eccB_5_-eccC_5_* genes were deleted or expressed under the control of an anhydrotetracycline-repressible promoter, we demonstrate that an intact *eccB_5_-eccC_5_* locus is essential for *M. tuberculosis* and that disruption/repression of single core components of the ESX-5 secretion machinery strongly impacts the *M. tuberculosis in vitro* growth properties. Taken together, the results obtained demonstrated the importance of an intact and functional ESX-5 for *M. tuberculosis* viability, emphasizing the key role of this secretion system in the biology of this human pathogen.

## Materials and Methods

### Bacterial Strains, Media and Growth Conditions

*Escherichia coli* strain DH10B (Stratagene), used for cloning procedures, was grown in LB broth (Sigma) or LB agar (Sigma). *Mycobacterium tuberculosis* H37Rv (stocks obtained from the Institut Pasteur) [22] was used as reference strain and for construction of deletion/conditional mutants. Mycobacterial strains were grown in Middlebrook 7H9 broth (Difco) supplemented with 10% (v/v) albumin-dextrose-catalase (ADC), 0.2% (v/v) glycerol, and 0.05% (v/v) Tween80 (Sigma) or on solid Middlebrook 7H11 medium (Difco) supplemented with 10% (v/v) oleic acid-albumin-dextrose-catalase (OADC). When required, the media were supplemented with 50 µg/ml kanamycin, 100 µg/ml hygromycin or 20 µg/ml gentamycin for *E. coli*, and 20 µg/ml kanamycin, 50 µg/ml hygromycin or 20 µg/ml streptomycin for *M. tuberculosis*.

For analysis of *in vitro* growth of conditional mutants, mycobacterial strains in exponential growth phase were diluted to an optical density at 600 nm (OD_600_) of 0.5, and 5 µl of 10-fold serial dilutions were spotted on Middlebrook 7H11 medium, added or not with different concentrations of anhydrotetracycline (ATc) (100, 200 and 400 ng/ml). Alternatively, bacterial strains were grown in Middlebrook 7H9 medium containing or not 400 ng/ml ATc. After 3 days, cultures were diluted to OD_600_ = 0.04 in fresh medium containing or not 400 ng/ml ATc, and the bacterial growth was monitored by daily OD_600_ measurements.

### RNA Extraction and Reverse Transcription RT- PCR/5′ RACE Assays

RNA extraction was performed from *M. tuberculosis* H37Rv cultures in exponential growth phase as previously described [24]. Briefly, bacteria were recovered by centrifugation and broken in 1 ml of TRIzol (Applied Biosystems) in presence of zirconia beads (0.1 mm diameter) in a MM300 apparatus (Qiagen) (30 sec at maximum speed). RNA was obtained by extraction with 0.2 vol of chloroform and precipitation for 1 h at –80°C with 0.1 vol of 3 M sodium acetate and 0.45 vol of isopropanol. Removal of contaminating DNA was performed using DNA*free* kit (Applied Biosystems/Ambion), according to the manufacturer’s instructions. RT-PCR reactions were performed using *Superscript one-step RT-PCR kit* (Applied Biosystems) as recommended by the producer. Sequences of primers used in amplification reactions are listed in Table S1. 5′ Rapid Amplification of cDNA Ends (RACE) was performed using the 5′/3′ RACE kit (Roche Molecular Biochemicals). One microgram of RNA and *eccB_5_*-specific primer (Table S1) were incubated at 70°C for 5 min. Denatured RNA and primer were then incubated at 55°C for 1 h in the presence of 1X cDNA synthesis buffer, 1 mM dNTPs, 40 U Protector RNase Inhibitor and 25 U Transcriptor Reverse Transcriptase. The cDNA obtained was purified by using the High Pure PCR Product Purification Kit (Roche Molecular Biochemicals), and used in poly(A) tailing reaction (30 min at 37°C in the presence of 0.2 mM dATP and 80 U Terminal Transferase). Nested PCR amplification on poly(A)-tailed cDNA was performed using an oligo dT-anchor primer and an *eccB_5_* specific primer (Table S1). The single amplification product obtained was directly sequenced.

### Construction of *eccB_5_*-*eccC_5_* Deletion and Conditional Mutants in *M. tuberculosis*

Deletion of the *eccB_5_*-*eccC_5_* segment in *M. tuberculosis* H37Rv was performed by allelic exchange using the ts-*sacB* technology [25]. Briefly, a 2445 bp and a 1227 bp fragment encompassing the *eccB_5_*-upstream region and the *eccC_5_*-downstream region, respectively, were amplified by PCR from *M. tuberculosis* H37Rv genomic DNA (See Table S1 for primer details). The amplicons were digested with *Spe*I/*Xba*I and *Xba*I/*Not*I, respectively, and cloned into the *Spe*I/*Not*I-digested pPR27 vector to obtain pMDL92. The *aph* cassette, conferring resistance to kanamycin, was amplified by PCR from the pUC4K plasmid, digested by *Xba*I and inserted into the *Xba*I-digested pMDL92 plasmid. The resulting plasmid, pMDL92-aph, was used in allelic exchange experiments. Kanamycin-resistant/sucrose-resistant (Kana^r^/Sac^r^) transformants were screened by PCR using primers specific for the *aph* cassette and for the *rv1780* or *rv1786* genes (see Table S1 for primer sequences).

The Mtb::*eccB_5_*-*eccC_5_* merodiploid strain was constructed by using the integrative vector pRBexint [26]. The genomic segment *eccB_5_*-*eccC_5_* was amplified by PCR (see Table S1 for primer details), digested with *Spe*I*/Hpa*I and cloned into the *Spe*I*/Hpa*I digested-pRBexint. The resulting plasmid (pExint*eccB_5_*-*eccC_5_*) was used to transform *M. tuberculosis*, and hygromycin resistant clones were selected. To construct *eccB_5_* and *eccC_5_* conditional mutants the recently developed TetR/Pip OFF mycobacterial repressible system was used [27], [28]. Briefly, a recombinant *M. tuberculosis* strain (Mtb::*tetR*-*pip*) was constructed, in which the genes encoding the tetracycline-sensitive repressor TetR and the *Streptomyces pristinaespiralis* Pip repressor, as well as the reporter gene *lacZ* were integrated into the genome at the *attB* site. Such a genomic organization allows the transcriptional repression of genes expressed under the control of the *P_ptr_* promoter as a consequence of the addition of ATc to the medium [27], [28]. The functionality of the PiP ON/Tet OFF regulatory circuit was confirmed by ß-galactosidase activity assays performed on the Mtb::*tetR*-*pip* strain, demonstrating that the addition of ATc to the culture medium results in a dose-dependent reduction of the expression of the *lacZ* gene reporter (Figure S1).

A 950-bp and 876-bp fragment encompassing the 5′-portions of *eccB_5_* and *eccC_5_* genes, respectively, were amplified by PCR on *M. tuberculosis* genomic DNA (see Table S1 for primer details), digested by *Nsi*I and cloned in frame with the *S. pristinaespiralis* promoter *P_ptr_*, into the *Nsi*I site of suicide vector pFRA50 [27]. The two obtained plasmids, pMDL-*eccB_5_* and pMDL-*eccC_5_* were then electroporated in the Mtb::*tetR*-*pip* strain, and recombinant clones were selected for resistance to hygromycin. Genomic DNAs from hygromycin resistant clones were analyzed by PCR for the correct integration of the *P_ptr_* promoter immediately upstream the *eccB_5_* or *eccC_5_* coding regions, respectively. Sequences of primers used in screening PCR reactions are reported in Table S1.

### Infection of THP-1 Human Macrophage Cell Line

THP-1 cells were cultured in RPMI medium supplemented with 10% heat-inactivated fetal calf serum (Euroclone) and 2 mM L-glutamine (Euroclone). Before infection, cells were seeded in 96-well plates at a density of 7.5×10^4^ and differentiated into macrophages by incubation with 50 nM PMA for 1 day. The Mtb*_Pptr_eccB_5_-eccC_5_* and Mtb*_Pptr_eccC_5_* conditional mutants as well as the *M. tuberculosis* control strain were pre-grown for 48 h in ATc-containing medium, and used in cell infection assays at a multiplicity of infection (m.o.i.) of 1∶20 (bacteria:cells). After phagocytosis (90 min), infected cells were washed twice with PBS to remove extracellular bacteria, and incubated for 6 days in culture media supplemented or not with 400 ng/ml ATc. Culture media, containing or not ATc, were replaced every 48 h as described elsewhere [27]. At different time points (immediately after phagocytosis, and 2, 4 and 6 days post infection), cells were lysed in PBS 0.01% Triton X-100 and the number intracellular bacteria was determined by plating 10-fold serial dilutions of cell lysates on solid medium.

### Preparation of Culture Supernatants and Total Lysates, SDS-PAGE and Immunoblotting

Conditional mutants and *M. tuberculosis* control strain were grown in Middlebrook 7H9 medium as described above. Pre-cultures in exponential growth phase were diluted to OD_600_ 0.04 in Middlebrook 7H9 fresh medium added with 0.1% (v/v) ADC, and containing or not 400 ng/ml ATc. After 3 and 6 days, culture supernatants were recovered and proteins were precipitated with 10% (w/v) TCA as previously described [19]. To obtain total lysates, mycobacterial pellets were washed twice and resuspended in 20 mM TrisCl. Bacterial cells were broken by shaking with acid washed-glass beads (106 µm diameter) for 8 min, in a Tissue Lyser apparatus (Qiagen). Suspensions were centrifuged at 5,000 rpm at 4°C for 30 min and the supernatant fraction obtained represented the total-cell lysate. Immunoblot analyses were performed with rabbit anti-EsxN [20] and anti-PPE41 [16] polyclonal sera, or anti-GroEL2 mouse monoclonal antibody (Colorado State University, NIH, NIAID contract NO AI75320) as previously reported [20].

## Results

### Characterisation of the *eccB_5_-eccC_5_* Locus in *M. tuberculosis* H37Rv

**Figure 1:**
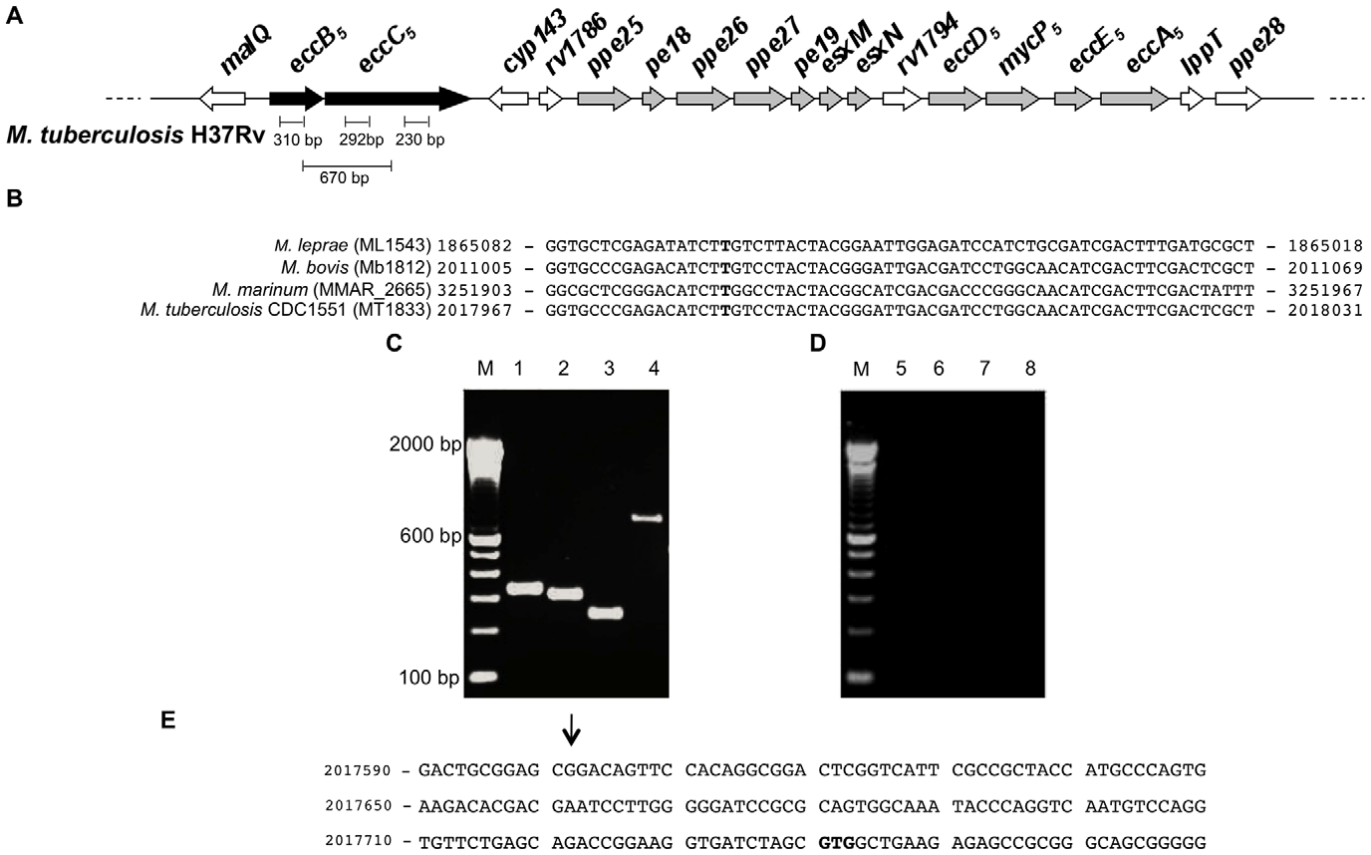
The *eccB_5_*-*eccC_5_* operon in *M. tuberculosis* H37Rv. (**A**) Schematic representation of chromosomal organization of ESX-5 locus in *M. tuberculosis* H37Rv. Black arrows represent *eccB_5_* and *eccC_5_* genes, while gray arrows indicate other ESX-5 genes coding for components of the corresponding secretory apparatus. White arrows represent ESX-5-flanking genes or region-associated genes coding for proteins not thought to be involved in the ESX-5 secretion machinery. Amplification fragments obtained in RT-PCR reactions are also depicted. (**B**) Comparison of *eccC_5_* sequence in different mycobacterial species and in the *M. tuberculosis* CDC1551 strain. The *eccC_5_* gene segment that includes the T (in bold) corresponding to the A at position 2020563 in the previously reported H37Rv reference genome sequence is represented. For each mycobacterial species or strain, numbers indicate the nucleotidic position in the corresponding annotated genome. (**C**) Analysis of *eccB_5_* and *eccC_5_* transcripts performed by RT-PCR on total RNA using various combinations of gene specific primers: primers specific for *eccB_5_* (lane 1); primers specific for the 5′ terminus of *eccC_5_* (lane 2); primers specific for the internal portion of *eccC_5_* (lane 3); primer specific for *eccB_5_* and *eccC_5_* (lane 4) M: molecular weight markers. (**D**) Control PCR reactions performed on RNA samples using the same combinations of *eccB_5_*- and *eccC_5_* specific primers. No amplification products were detected, thus confirming the absence of contaminating genomic DNA in RNA preparations. M: molecular weight markers. (**E**) DNA sequence of the *eccB_5_* upstream region. The 5′ end of the *eccB_5_-eccC_5_* transcript is indicated by the arrow. The *eccB_5_-eccC_5_* translational start site is indicated in bold. Numbers indicate the nucleotidic position in the annotated genome of *M. tuberculosis* H37Rv strain.

EccB_5_ and EccC_5_ are encoded by genes *eccB_5_* (*rv1782*) and *eccC_5_* (*rv1783-rv1784*) located in the 5′ region of the ESX-5 locus of *M. tuberculosis* (Figure 1A). As for the ESX-1-associated EccC_1_, which is encoded by the two adjacent genes *eccCa_1_* (*rv3870*) and *eccCb_1_* (*rv3871*), EccC_5_ was thought to be encoded by two separate genes, *eccCa_5_* (*rv1783*) and *eccCb_5_* (*rv1784*) in the *M. tuberculosis* H37Rv reference strain [7], [22]. Conversely, it was reported to be encoded by a single non-divided *eccC_5_* gene in several mycobacterial species such as *Mycobacterium leprae*, *Mycobacterium bovis* and *Mycobacterium marinum,* as well as in other *M. tuberculosis* strains (Figure 1B) [29]–[32]. Sequence verification of the *M. tuberculosis* H37Rv strain used in our study revealed that the in frame stop codon separating *eccC_5_* gene into two genes, previously reported in the H37Rv reference genome sequence [7], [22], seems to be due to a sequencing error (A instead of T at position 2020563) (Figure S2). Thus, in agreement with sequence data reported for other *M. tuberculosis* H37Rv variants [32], a single *eccC_5_* is also present in the *M. tuberculosis* H37Rv strain used in this study (Figure 1A).

The genomic organisation of *eccB_5_*/*eccC_5_* locus (Figure 1A) suggested that the two genes might be cotranscribed and form a transcriptional unit independent from their flanking genes. Analysis of *eccB_5_*/*eccC_5_* transcripts performed by RT-PCR using different combinations of *eccB_5_*/*eccC_5_* gene-specific primers revealed a 670-bp amplicon when a combination of primers specific for *eccB_5_* and *eccC_5_* was used (Figure 1C, lane 4), thus indicating that these two genes are indeed cotranscribed. Transcription data were confirmed by mapping the 5′ end of the *eccB_5_*-*eccC_5_* transcript using 5′ RACE, which identified only one specific transcript starting 139 bases upstream of the *eccB_5_* start codon (Figure 1E).

### The *eccB_5_-eccC_5_* Operon is Essential for *in Vitro* Growth of *M. tuberculosis*

**Figure 2:**
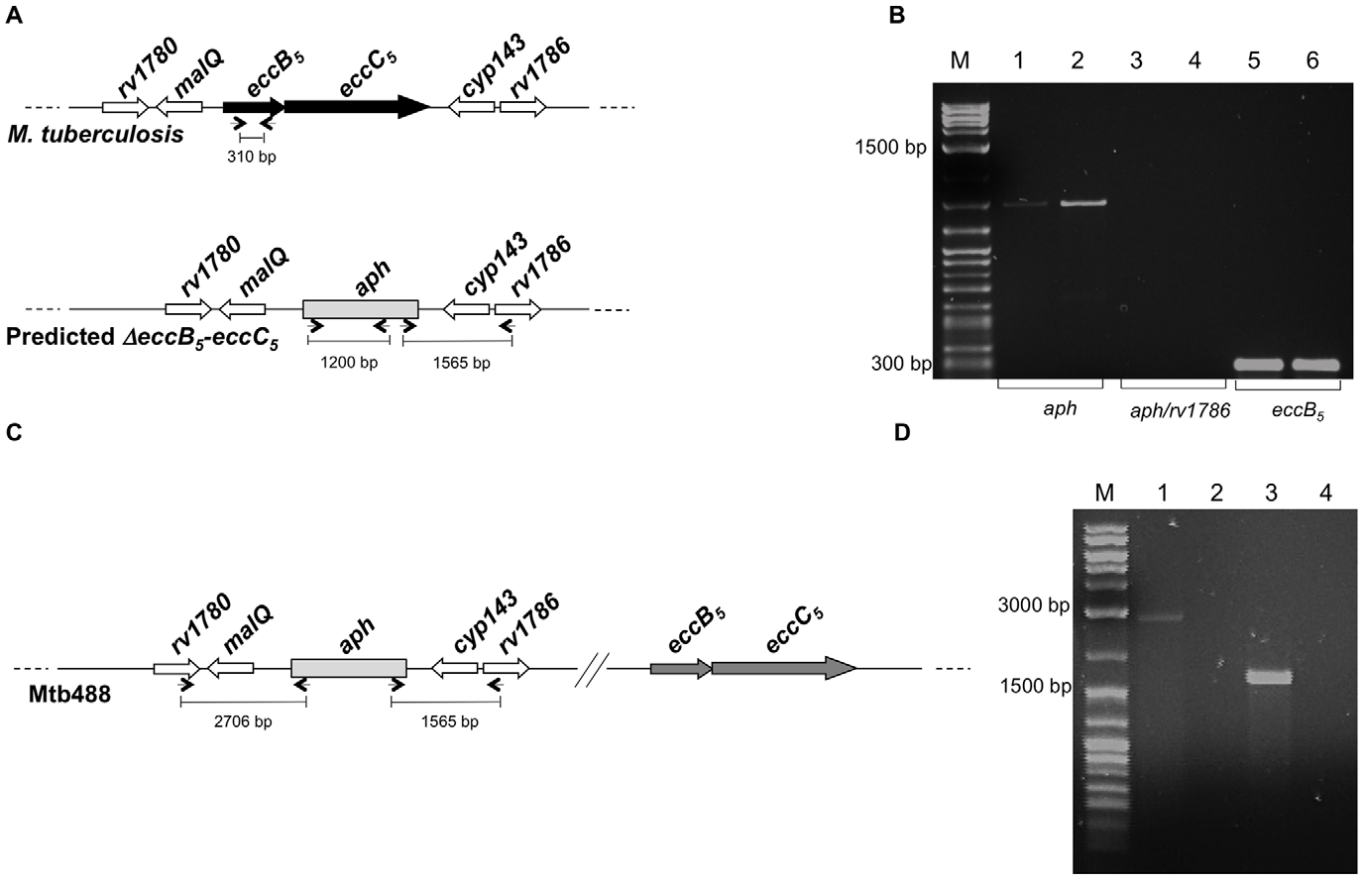
Deletion of the *eccB_5_*-*eccC_5_* operon in *M. tuberculosis.* (**A**) Predicted genomic organization of a putative *M. tuberculosis* mutant, in which the *eccB_5_-eccC_5_* operon is deleted and replaced with an *aph* cassette. (**B**) Amplification profiles of genomic DNAs from two representative kan^r^/suc^r^ clones, obtained in PCR reaction carried out by using primers specific for the *aph* cassette and *eccB_5_-eccC_5_* flanking genes (not included in the plasmid construct used in allelic exchange experiments), namely *rv1780* (lanes 3–4) and *rv1786* (data not shown). In these reactions, amplification products were expected only if the *eccB_5_-eccC_5_* operon at the ESX-5 locus is replaced by the *aph* cassette; in contrast, no amplification products were expected in case of non-homologous recombination. PCR control reactions were performed using primers specific for the *aph* cassette (lanes 1–2) or the *eccB_5_* gene (lanes 5–6). (**C**) Genomic organization of Mtb488, a representative mutant deleted for the *eccB_5_*-*eccC_5_* operon in the Mtb::*eccB_5_*-*eccC_5_* genetic background. The gray box represents the *aph* cassette replacing the *eccB_5_-eccC_5_* operon at the ESX-5 locus; dark gray arrows indicate the additional copies of *eccB_5_* and *eccC_5_* genes integrated at the *attB* site. (**D**) PCR profiles of genomic DNA from Mtb488 (lanes 1 and 3) amplified with primers specific for the *aph* cassette and *rv1780* gene (lane 1) or the *aph* cassette and *rv1786* gene (lane 3). Again, these PCR reactions were expected to give amplification products only if the native *eccB_5_-eccC_5_* operon was replaced by the *aph* gene. As expected, no amplification products were detected in PCR reactions performed/carried out on genomic DNA from wild-type H37Rv *M. tuberculosis* (lanes 2 and 4), which was used as negative control.

Insertional mutagenesis and transposon site hybridization (TraSH) studies performed in *M. tuberculosis* identified transposon insertion mutants in various genes coding for different components of the ESX-5 secretion apparatus, such as *eccA_5_* or *eccD_5_*, but no consistent data were available for *eccB_5_*-*eccC_5_* [33], [34]. To assess the potential essentiality of the *eccB_5_*-*eccC_5_* operon, an attempt to delete both genes was performed by using a classical knockout strategy based approach, which employs the replicative thermosensitive vector pPR27, carrying the *sacB* counterselectable marker [25]. A recombinant pPR27 plasmid containing the *aph* cassette encoding resistance to kanamycin flanked by *eccB_5_*-upstream and *eccC_5_*-downstream regions was constructed and used in the allelic replacement experiments. According to this strategy, at the end of the procedure putative *M. tuberculosis* double cross over recombinants with a kan^r^/suc^r^ phenotype were expected to carry the *aph* cassette replacing the *eccB_5_-eccC_5_* operon. However, PCR analysis of genomic DNAs from 150 clones, using primers specific for the *aph* cassette and r*v1780* or *rv1786* genes, revealed that none of them showed the expected pattern for an allelic exchange mutant (Figure 2 A and B). While *eccB_5_*- and *aph* specific amplification products were detected in PCR control reactions (Figure 2B, lanes 1–2 and 5–6, respectively), no *aph-rv1786* specific amplification products were obtained (Figure 2B, lanes 3–4), demonstrating the presence of an intact *eccB_5_-eccC_5_* operon at the ESX-5 locus, as well as the non homologous integration of the *aph* cassette into the genomes of the tested kan^r^/suc^r^ clones. As in a previous work the same technique has allowed the deletion of genomic segments up to ∼ 20 kb (in size) in *M. tuberculosis* [24], the inability to obtain *eccB_5_-eccC_5_* knock-out mutants in this study strongly suggests that the deletion of the *eccB_5_-eccC_5_* operon might be lethal for *M. tuberculosis*.

To confirm the requirement of an intact *eccB_5_-eccC_5_* operon for growth of *M. tuberculosis* an Mtb::*eccB_5_-eccC_5_* merodiploid strain, carrying an additional wild-type copy of *eccB_5_-eccC_5_* genes integrated into the *attB* site of the genome, was constructed. Allelic exchange experiments were then repeated in the Mtb::*eccB_5_-eccC_5_* merodiploid genetic background. Again, genomic DNAs from resultant kan^r^/suc^r^ clones were tested by PCR, using combinations of primers specific for the *aph* cassette and *rv1780* or *rv1786* genes. As depicted in Figure 2D for a representative mutant (Mtb488), the presence of 2706-bp *aph-rv1780-* specific and 1585-bp *aph-rv1786-* specific amplification products confirmed the replacement of the wild-type *eccB_5_*-*eccC_5_* genomic segment at the ESX-5 locus with the *aph* cassette. PCR analysis revealed that the deletion of *eccB_5_-eccC_5_* genes at the ESX-5 locus had occurred in 5% of the recombinants, thus demonstrating that only the presence of an additional copy of *eccB_5_-eccC_5_* genes allowed the deletion of the chromosomal *eccB_5_-eccC_5_* segment to take place.

### Construction of Mtb*_Pptr_eccB_5_* and Mtb*_Pptr_eccC_5_* Conditional Mutant Strains

**Figure 3:**
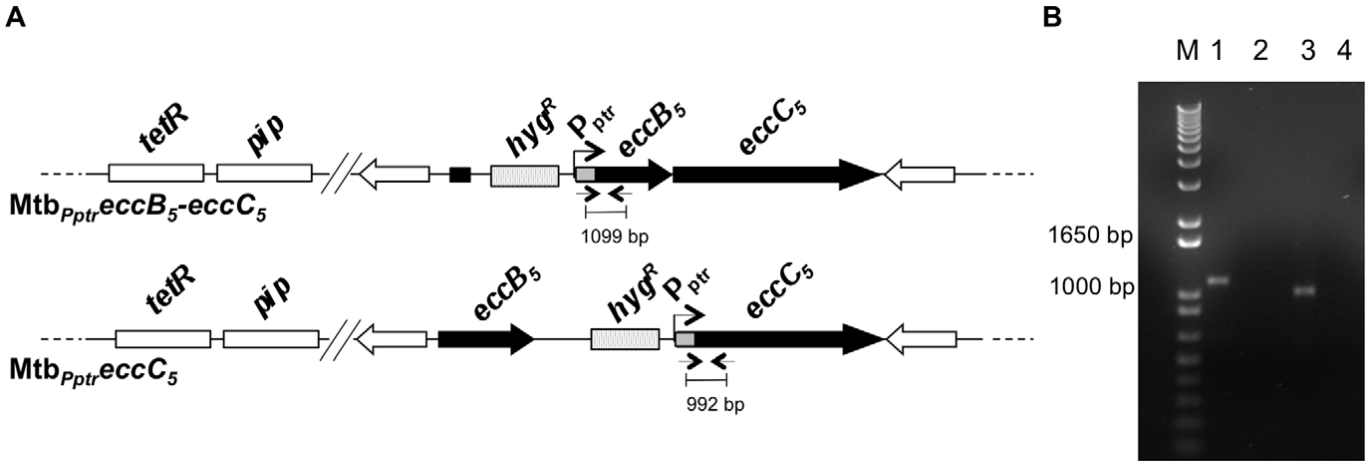
Construction of Mtb*_Pptr_eccB_5_-eccC_5_* and Mtb*_Pptr_eccC_5_* conditional mutants. (**A**) Schematic representation of genomic organization of Mtb*_Pptr_eccB_5_-eccC_5_* and Mtb*_Pptr_eccC_5_* strains. White boxes represent the TetR and the Pip encoding genes integrated at the *attB* site of the genomes of Mtb*_Pptr_eccB_5_-eccC_5_* and Mtb*_Pptr_eccC_5_* conditional mutants; white arrows indicated the ESX-5 region-associated genes flanking the *eccB_5_-eccC_5_* operon; dotted boxes represent the hygromycin resistance marker; black arrows indicate the *eccB_5_* and the *eccC*_5_ genes at ESX-5 locus; gray boxes represent the *P_ptr_* promoter integrated immediately upstream the *eccB_5_* or the *eccC_5_* coding region in the Mtb*_Pptr_eccB_5_-eccC_5_* and Mtb*_Pptr_eccC_5_* strain, respectively. Arrows represent primers used for PCR analysis. (**B**) Analysis of genomic DNA from Mtb*_Pptr_eccB_5_-eccC_5_* (lane 1) and Mtb*_Pptr_eccC_5_* (lane 3) by PCR using primers specific for the *P_ptr_* promoter and an internal region of *eccB_5_* or *eccC_5_* genes, respectively. PCR reactions were also performed on genomic DNA form wild-type *M. tuberculosis* (lane 2 and 4), which was used as negative control. Amplification profiles obtained demonstrated the correct integration of the *P_ptr_* promoter upstream the *eccB_5_* or *eccC_5_* coding regions, in the Mtb*_Pptr_eccB_5_-eccC_5_* and Mtb*_Pptr_eccC_5_* mutants, respectively.

To investigate more in depth the effect of inactivation of each gene of the *eccB_5_-eccC_5_* locus on growth of *M. tuberculosis*, two conditional mutant strains were constructed using the Pip ON/Tet OFF repressible system [27]. This system, which has been successfully used to construct and characterize an *M. tuberculosis* conditional mutant for the ESX-3 secretion system [28], is based on the expression of the gene of interest under the control of the anhydrotetracycline-repressible *P_ptr_* promoter [27]. As schematically depicted in Figure 3A, in the first mutant, the *P_ptr_* promoter is placed immediately upstream of the *eccB_5_* coding sequence, replacing the physiological *eccB_5_* promoter. As *eccB_5_* and *eccC_5_* genes are cotranscribed from the same promoter (Figure 1 C and E), this mutant is actually a conditional mutant for the *eccB_5_-eccC_5_* operon and was thus referred as Mtb*_Pptr_eccB_5_-eccC_5_*. In the second mutant (Mtb*_Pptr_eccC_5_*), the *P_ptr_* promoter is placed immediately upstream the *eccC_5_* coding region, so that only the *eccC_5_* is regulated by *P_ptr_*, while the *eccB_5_* gene is expressed from its own promoter. Both conditional mutants are derivatives of the *Mtb::tetR-pip* strain and carry the tetracycline-sensitive repressor TetR and the Pip repressor encoding genes integrated into the chromosome at the *attB* site. A 950-bp and 876-bp fragment encompassing the 5′-portions of *eccB_5_* and *eccC_5_* coding regions, respectively, were cloned in frame with the *P_ptr_* promoter into the suicide vector pFRA50, and recombinant plasmids obtained were used to transform the *Mtb::tetR-pip* strain. Analysis by PCR on genomic DNA from selected hygromycin resistant clones confirmed the correct replacement of the *eccB_5_-eccC_5_* promoter with the *P_ptr_* promoter in the Mtb*_Pptr_eccB_5_-eccC_5_* strain, and the insertion of the *P_ptr_* promoter upstream the *eccC_5_* gene in the Mtb*_Pptr_eccC_5_* mutant (Figure 3B). In these strains, the expression of *eccB_5_-eccC_5_* operon or the *eccC_5_* gene is exclusively regulated by the activity of the *P_ptr_* promoter, which in turn is strictly dependent on the absence/presence of ATc in the culture medium (Figure S3). In the absence of ATc, TetR binds to its operators, turning off the *pip* transcription, and thus allowing the expression of the *eccB_5_-eccC_5_* or the *eccC_5_* genes from the *P_ptr_* promoter (Figure S3 A). In the presence of ATc, the transcription of *pip* is allowed. The Pip production results in the block of the *P_ptr_* promoter activity, and finally in the repression of *eccB_5_-eccC_5_* or *eccC_5_* gene expression (Figure S3 B).

### Repression of *eccB_5_-eccC_5_* Shows Stronger Impact on *in vitro* Growth of *M. tuberculosis* than Repression of *eccC_5_*

**Figure 4:**
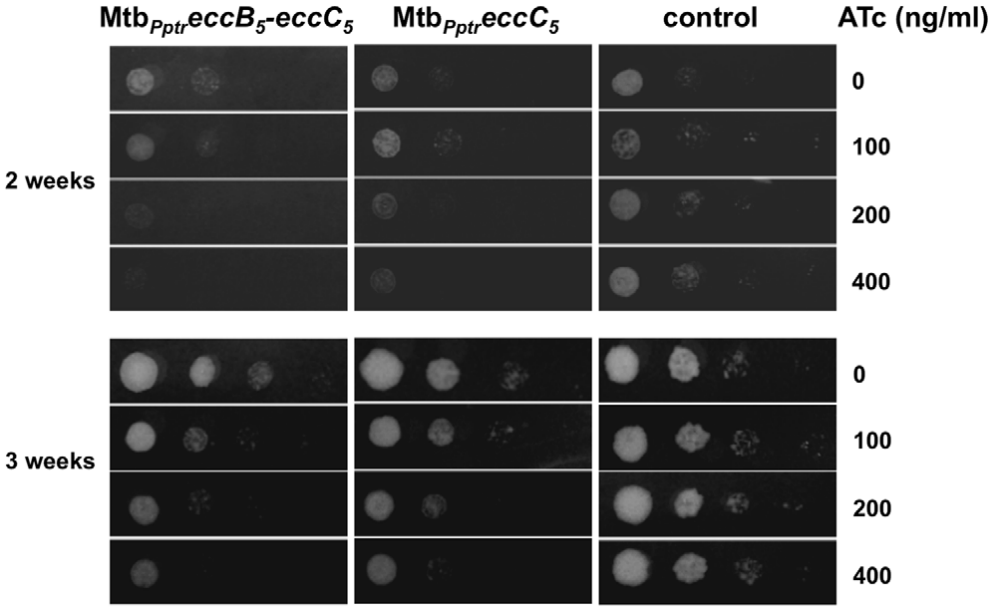
Growth of Mtb*_Pptr_eccB_5_-eccC_5_* and Mtb*_Pptr_eccC_5_* strains on solid medium. Ten-fold serial dilutions of cultures from Mtb*_Pptr_eccB_5_-eccC_5_*, Mtb*_Pptr_eccC_5_* and *M. tuberculosis* control strain (Mtb::*tetR-pip*) were plated on Middlebrook 7H11, containing different concentrations of ATc, ranging from 0 to 400 ng/ml. Bacterial growth was checked after 2 and 3 weeks of incubation at 37°C.

The impact of EccB_5_ and EccC_5_ on *M. tuberculosis* viability was determined by evaluating the growth properties of Mtb*_Pptr_eccB_5_-eccC_5_* and Mtb*_Pptr_eccC_5_* conditional mutants both on solid and liquid media. Ten-fold dilutions of Mtb*_Pptr_eccB_5_-eccC_5_* and Mtb*_Pptr_eccC_5_* mutants or *M. tuberculosis* control strain were spotted onto Middlebrook 7H11 medium added with different concentrations of ATc, ranging from 100 to 400 ng/ml. As control, bacterial dilutions were also spotted on the same medium without the antibiotic. While the growth of the control strain was not affected by the presence of ATc even at the highest concentration of the antibiotic, the growth of the Mtb*_Pptr_eccB_5_-eccC_5_* mutant was strongly affected by the presence of the antibiotic in an ATc-concentration dependent manner (Figure 4). After 2 weeks of incubation, no growth was indeed observed for the Mtb*_Pptr_eccB_5_-eccC_5_* strain on plates containing the highest concentration of ATc, and some colonies were detectable for the mutant only on agar plates containing lower ATc concentrations. After 3 weeks of incubation, whereas bacterial growth was detected for the control strain at higher concentrations of antibiotic and less concentrated bacterial dilutions, some growth was detectable for the Mtb*_Pptr_eccB_5_-eccC_5_* mutant, but only at the most concentrated bacterial dilutions.

**Figure 5:**
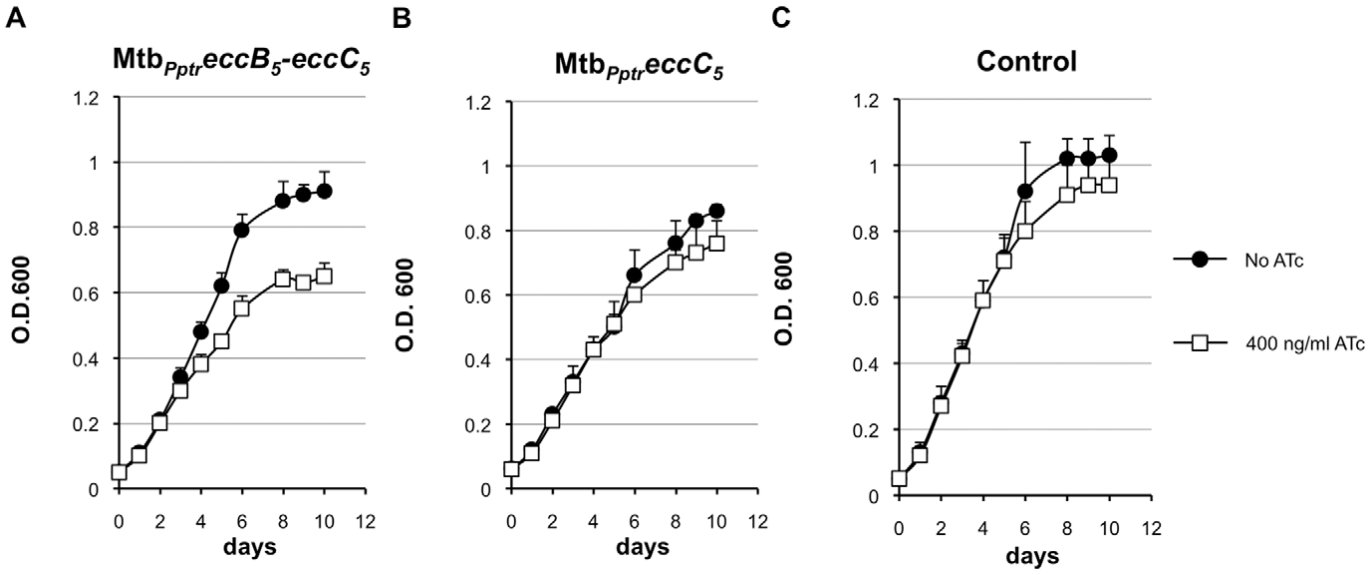
Growth of Mtb*_Pptr_eccB_5_-eccC_5_* and Mtb*_Pptr_eccC_5_* mutants in liquid medium. Mtb*_Pptr_eccB_5_-eccC_5_* (**A**), Mtb*_Pptr_eccC_5_* (**B**) and *M. tuberculosis* control strain (Mtb::*tetR-pip*) (**C**) were grown in Middlebrook 7H9 medium with or without 400 ng/ml ATc. Bacterial growth was monitored by daily measurements of OD_600_. The figure shows the mean and the standard deviations of OD_600_ values/measurements obtained in three independent experiments.

Similar results were obtained when the growth kinetics of the Mtb*_Pptr_eccB_5_-eccC_5_* conditional mutant and control strain were analyzed in liquid medium (Figure 5). Again, the growth of the *M. tuberculosis* control strain was not affected by the addition of ATc into the medium (Figure 5C). In contrast, the growth of Mtb*_Pptr_eccB_5_-eccC_5_* mutant was impaired in the presence of 400 ng/ml ATc (Figure 5A), a growth defect that was detectable after 3 days from the addition of the ATc. As EccB_5_ and EccC_5_ are predicted to be structural components of the *M. tuberculosis* ESX-5 membrane complex it is possible that EccB_5_ and EccC_5_ proteins already produced by bacterial strains before the exposure to ATc might compensate the effect of *eccB_5_-eccC_5_* gene repression immediately after the addition of the antibiotic. These results further confirm that the repression of the *eccB_5_-eccC_5_* operon strongly inhibits the *M. tuberculosis in* *vitro* growth.

The Mtb*_Pptr_eccC_5_* strain, in which only the *eccC_5_* gene is repressed in presence of ATc, showed a different phenotype. This mutant displayed impaired growth as compared to the control strain on agar plates containing ATc (Figure 4), indicating that the repression of *eccC_5_* affects the ability of *M. tuberculosis* to grow on solid medium. In contrast, no difference between the Mtb*_Pptr_eccC_5_* mutant and the control strain was detected when the growth was evaluated in liquid medium (Figure 5). In this case, similarly to the *M. tuberculosis* control strain, the growth of EccC_5_ conditional mutant was not affected by the addition of ATc, and comparable growth kinetics were observed for the Mtb*_Pptr_eccC_5_* strain in Middlebrook 7H9 medium added or not with the antibiotic marker (Figure 5B). Taken together with the results from knock-out experiments these data clearly emphasize that the *eccB_5_-eccC_5_* operon is essential for *M. tuberculosis* viability.

### *eccB_5_-eccC_5_* and *eccC_5_* Repression Impairs the Growth of *M. tuberculosis* in THP-1 Derived Macrophages

**Figure 6:**
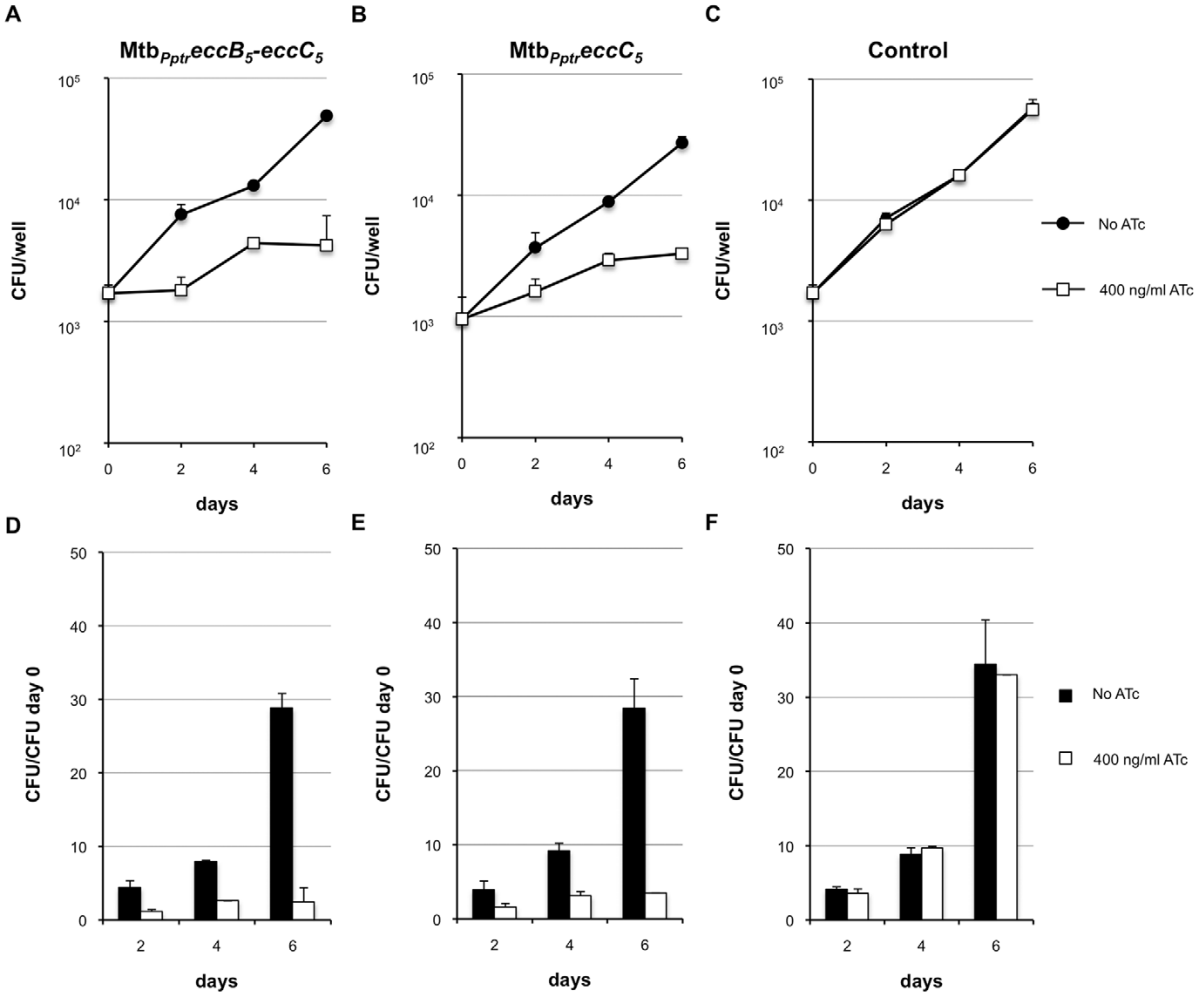
Intracellular growth kinetics of Mtb*_Pptr_eccB_5_-eccC_5_* and Mtb*_Pptr_eccC_5_* strains in THP-1 derived macrophages. THP-1-derived macrophages were infected with Mtb*_Pptr_eccB_5_-eccC_5_* and Mtb*_Pptr_eccC_5_* conditional mutants as well as with the *M. tuberculosis* control strain at a m.o.i of 1∶20 (bacteria:cells), and cultured in the presence or not of ATc. Immediately after phagocytosis and 2, 4 and 6 days after infection, the number of viable intracellular bacteria was determined. The figure reports the means of CFU number (A, B, and C) and CFU ratio values (CFU/CFU at day 0) (D, E and F) obtained in a representative experiment performed in triplicate.

Inactivation of single ESX-5-associated genes encoding building blocks of the ESX-5 secretion apparatus (e.g. *eccD_5_*) results in the loss of the ability of *M. tuberculosis* to replicate in *ex vivo* models [20]. To assess the impact of *eccB_5_-eccC_5_* and *eccC_5_* repression on the *M. tuberculosis* intracellular growth properties the growth kinetics of Mtb*_Pptr_eccB_5_-eccC_5_* and Mtb*_Pptr_eccC_5_* conditional mutants were compared in THP-1-derived macrophages. Cells were infected with different strains at a m.o.i of 1∶20 (bacteria:cells) and cultured in presence or not of 400 ng/ml ATc. At these concentrations, ATc is not toxic for THP-1, as evaluated by Alamar blu assay (data not shown), but can penetrate into the cells and down regulate the expression of *P_Ptr_* -controlled genes [27]. At different times post infection (after phagocytosis and at day 2, 4 and 6), the number of viable intracellular bacteria was determined. Results obtained are depicted in Figure 6. While the intracellular growth of the control strain was not affected by the presence of the antibiotic in the culture medium (Figure 6 C and F), the growth of the *eccB_5_-eccC_5_* mutant was strongly inhibited in presence of ATc (Figure 6 A and D). At day 6 post infection, the CFU ratio (CFU/CFU at day 0) value for this mutant was 2.4 in THP-1 macrophages cultured in medium containing ATc, whereas it was 28.8 in cells incubated in the absence of the antibiotic (Figure 6 D). A similar attenuation was observed for the *eccC_5_* mutant (Figure 6 B and E), for which CFU ratio values were 3.5 and 28.4 in THP-1 macrophages cultured in presence or in absence of ATc, respectively (Figure 6 E). These data demonstrate that expression of *eccB_5_* and *eccC_5_* is required for optimal replication of *M. tuberculosis* in macrophages.

### EccB_5_ and EccC_5_ are Required for Secretion of ESX-5 specific Substrates

**Figure 7:**
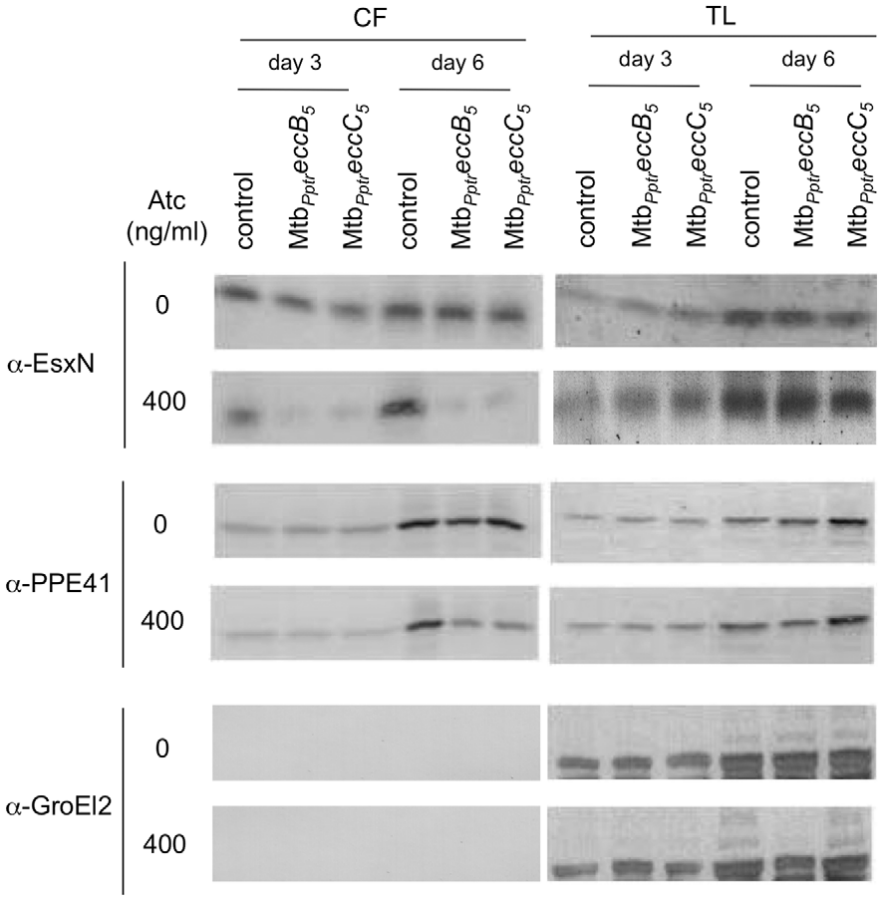
EsxN and PPE41 secretion in Mtb*_Pptr_eccB_5_-eccC_5_* and Mtb*_Pptr_eccC_5_*. Mtb*_Pptr_eccB_5_-eccC_5_* and Mtb*_Pptr_eccC_5_* conditional mutants as well as *M. tuberculosis* control strain (Mtb::*tetR-pip*) were grown in presence or absence of 400 ng/ml ATc. After 3 and 6 days, culture supernatants (CF) and total lysates (TL) were prepared and tested in Western blot with anti-EsxN or anti-PPE41 rabbit polyclonal sera. Culture supernatants from all strains were negative for GroEL2, thus indicating the absence of contamination of these samples with cytoplasm or cell wall associated proteins.

ESX-5 is responsible for secretion/transport of EsxN, the Esx protein encoded by the ESX-5 locus, and PPE41, a representative member of the large PPE protein family [20]. To determine the impact of EccB_5_ and EccC_5_ on secretion of ESX-5 specific substrates, the presence of EsxN and PPE41 in culture supernatants from Mtb*_Pptr_eccB_5_-eccC_5_* and Mtb*_Pptr_eccC_5_* strains grown in the presence or not of ATc was investigated. As expected, similar amounts of EsxN were detected in the culture supernatants from EccB_5_ and EccC_5_ mutants grown in the absence of ATc, as well as in samples from *M. tuberculosis* control strain (Figure 7). In contrast, while the addition of ATc did not affect the EsxN secretion in the control strain, the presence of the antibiotic abolished the secretion of the protein in EccB_5_ and EccC_5_ conditional mutants. Three and six days after addition of ATc to the medium, no EsxN was indeed detectable in the culture supernatants from Mtb*_Pptr_eccB_5_-eccC_5_* and Mtb*_Pptr_eccC_5_* strains, despite the presence of an EsxN-specific band in the corresponding total lysate samples (Figure 7). Similar results were obtained when the secretion of PPE41 was analyzed. Comparable amounts of the protein were detected in the culture supernatants recovered at day 6 from all mycobacterial strains grown in the absence of ATc (Figure 7). Again, while the addition of the antibiotic did not impact the secretion of PPE41 in the control strain, the presence of ATc into the medium resulted in a significant reduction of the amount of PPE41 exported in culture supernatants form the Mtb*_Pptr_eccB_5_-eccC_5_* and Mtb*_Pptr_eccC_5_* mutants (Figure 7).

Together these results confirmed that EccB_5_ and EccC_5_ are building blocks of the ESX-5 secretion machinery, both required for transport of ESX-5 specific substrates outside the cell.

## Discussion

In previous studies we demonstrated that selected ESX-5 encoded genes had an impact on secretion of ESX-5 specific substrates, cell wall integrity and virulence of *M. tuberculosis* [20], [21]. However, for some other ESX-5 associated genes, i.e. *eccB_5_/C_5_*, initial gene-inactivation attempts were unsuccessful, which made us think that these genes might be essential for *M. tuberculosis*. As results from TraSH analysis were inconclusive for this genomic locus [34], the impact of the *eccB_5_* and *eccC_5_* on *in vitro* growth of *M. tuberculosis* was thus studied in more detail. The ability to construct an *eccB_5_-eccC_5_* deletion mutant only in the presence of a second functional copy of *eccB_5_-eccC_5_* genes, as well as the inability of Mtb*_Pptr_eccB_5_-eccC_5_* (in which the *eccB_5_-eccC_5_* operon is repressed) to grow on solid medium consistently demonstrate that an intact *eccB_5_-eccC_5_* locus is essential for viability of *M. tuberculosis*. In a recent study by Griffin and colleagues, in which *M. tuberculosis* essential genes were identified by using high density transposon mutagenesis and next generation sequencing techniques, no transposon insertions were found in *rv1783* gene (encoding the N-terminal part of EccC_5_), whereas only a few insertions were detected in *rv1784* (encoding the C-terminal part of EccC_5_) or in *rv1782* (*eccB_5_*) [35], further indicating that, in accordance with our data, an intact *eccB_5_-eccC_5_* (*rv1782-rv1784*) locus is indispensable for growth of *M. tuberculosis*. The finding that the only ESX-5 *M. marinum* mutants identified in transposon mutagenesis studies were inactivated for MMAR_2676 and MMAR_2680/*eccA_5Mm_* genes (orthologous to the *M. tuberculosis rv1794* and *rv1798*/*eccA_5Mt_* genes, respectively) [16], [19], suggests that the ESX-5 locus in *M. marinum* might also encode some ESX-5 components that are essential for its *in vitro* growth. It seems clear that the inactivation of ESX-5 via the simultaneous deletion/repression of various genes encoding building blocks of the ESX-5 secretion machinery strongly affects the viability of *M. tuberculosis*, and might explain the reason why in this study it was not possible to obtain *M. tuberculosis* mutants deleted for the *eccB_5_-eccC_5_* operon. EccB_5_ and EccC_5_ are required for secretion of ESX-5 specific substrates (Figure 7) and are both predicted to encode membrane-bound ESX-5 components. During the preparation of this manuscript, the characterization of the composition of the ESX-5 membrane-bound complex in *M. marinum* and *M. bovis* BCG was reported [23]. Although the impact of *eccB_5_*-*eccC*_5_ inactivation on mycobacterial *in vitro* growth properties was not investigated, secretion and/or biochemical data confirmed the involvement of EccB_5_ and EccC_5_ as core components of the ESX-5 secretion apparatus.

ESX-5 has a strong impact in maintaining the mycobacterial cell wall integrity, and inactivation of a single core component of the ESX-5-transmembrane complex results in an increased sensitivity to detergents and hydrophilic antibiotics to which mycobacteria are naturally resistant [20]. It is possible that the disruption of large portions of the ESX-5 secretion apparatus, as is the case for the *eccB_5_-eccC_5_* deletion/repression, causes more extensive damage to the cell wall or more profound alterations to cell wall stability, resulting in the inability of the corresponding mutant strain to grow both on solid and liquid media. We also observed the intriguing phenomenon that inactivation of a single ESX-5 core component, such as EccC_5_, has an impact on the mutant’s growth characteristics on solid medium, whereas it has not any discernable inhibitory effect on growth in liquid medium. Interestingly, *eccC_5_* repression strongly affects the intracellular growth properties of *M. tuberculosis*, further confirming that repression of single genes encoding ESX-5 structural components is sufficient to strongly impair the growth of *M. tuberculosis* in a restrictive environment such as the macrophage. A similar phenotype has indeed been previously observed for a different ESX-5 mutant, in which the *eccD_5_* gene encoding the predicted ESX-5 transmembrane channel was disrupted [20]. The EccD_5_ mutant is unable to replicate in murine macrophages, displayed an impaired growth on solid medium, as revealed by a small colony morphotype on Middlebrook 7H11 agar plates, but showed no difference in growth as compared to the control strain in liquid medium, thus suggesting a potential role for the ESX-5 system in modeling the bacterial surface during colony formation on solid medium. A functional link between ESX secretion systems and mycobacterial cell wall has been demonstrated in *M. marinum*, where the ESX-1-encoded EccA_1_ ATPase, involved in secretion of ESX-1 substrates, was also found to be required for optimal synthesis of mycolic acids [36]. The recent finding that a plethora of genes encoding enzymes involved in cell-wall synthesis or functioning as well as various ESX loci (ESX-1, ESX-2 and ESX-5) are regulated by the nucleoid-associated EspR regulator [37], provides further evidence of the link between two mycobacterial virulence hallmarks such as ESX-mediated protein secretion/transport and cell envelop biogenesis.

In addition to the alterations of cell wall properties, it is plausible that such impairment in transport and secretion of ESX-5 specific substrates could contribute to the loss of viability observed after disruption of large portions of ESX-5. As a functional ESX-5 is required not only for secretion of PPE proteins [20], [21] but also for their correct localization in the cell wall [20], it cannot be excluded that the massive intracellular accumulation of un-secreted or incorrectly localized ESX-5 substrates can exert a toxic, lethal effect for mycobacterial cells. ESX-5 systems of various mycobacterial species have been demonstrated or predicted to be involved in transport of PE_PGRS [19], and a number of PE/PPE proteins encoded in and outside the ESX-5 locus [20], [21]. Consistent with previous data reported for another *M. tuberculosis* H37Rv ESX-5 knock-out strain [20], Western blot analyses of culture supernatants and cell lysates using a monoclonal antibody specific for the PGRS domain [19] did not reveal differences in the PE_PGRS profiles in samples from Mtb*_Pptr_eccB_5_-eccC_5_* or Mtb*_Pptr_eccC_5_* mutant strains, grown in the presence or not of ATc (data not shown). This finding thus argues against the possibility that the effects of *eccB_5_-eccC_5_* and *eccC_5_* repression on *M. tuberculosis* growth, as well as the phenotypic differences observed for *eccB_5_-eccC_5_* and *eccC_5_* mutants might be related to alterations/differences in secretion or intracellular accumulation of PE_PGRS proteins. On the other hand, information on the presence of ESX-5 associated PE/PPE proteins and their homologs encoded outside the ESX-5 locus in samples from *eccB_5_-eccC_5_* and *eccC_5_* mutants are not available, due to the lack of antibodies specific for these proteins. Thus, at present it cannot be excluded that the defect in *in vitro* growth observed for *eccB_5_-eccC_5_* or *eccC_5_* mutants might also be caused in part by the lack of transport and/or by the intracellular accumulation of these substrates (or some of them).

Although an intact ESX-5 system is required for optimal growth of *M. tuberculosis* and *M. marinum*, the ESX-5 locus is absent in the genomes of fast growing, saprophytic mycobacterial species [8]. These findings suggest that during the evolution of the slowly growing, pathogenic mycobacteria, duplication-diversification events have led to the emergence of ESX-5 systems that were apparently linked to the expansion of the PE/PPE protein family. It might well be that fast-growing mycobacteria, which possess only a very limited set of PE/PPE proteins, have no need for an ESX-5 secretion system, while slow-growers that harbor a wide range of PE/PPE proteins require a fully functional ESX-5 system that exports these proteins to the cell envelop and beyond for their viability. The described situation resembles observations made for the ESX-3 system involved in mycobactin-mediated iron uptake, which is essential in *M. tuberculosis* but dispensable for growth in the saprophytic species *M. smegmatis* [27], [38]. Furthermore, ESX-1 is a key virulence determinant in pathogenic mycobacteria but seems to regulate the DNA transfer in *M. smegmatis* [2], [39]. Such differences represent an intriguing and unexplored aspect of ESX secretion systems, leading to the requirement to study the individual ESX systems in the context of the mycobacterial species concerned. Because of their crucial role in host-pathogen interactions as well as their involvement in basic biological processes of tubercle bacilli, ESX secretion systems of *M. tuberculosis* thus represent potential targets for new anti-tuberculosis drugs directed against key mycobacterial factors required for viability and virulence.

## Supporting Information

Figure S1 Characterization of the TetR/Pip OFF system in the Mtb::*tetR-pip* strain. ß-galactosidase assay performed on Mtb::*tetR-pip* strain grown on Middlebrook 7H11 plates containing X-gal (40 µg/ml) and different concentrations of ATc, ranging from 0 to 200 ng/ml. The ß-galactosidase activity was clearly detected when bacteria were grown in the absence of ATc, and decreased in the presence of increasing concentrations of the antibiotic. The ß-galactosidase activity was abolished when Mtb::*tetR-pip* was grown on Middlebrook 7H11 medium containing 200 ng/ml ATc.
(TIF)

Figure S2 Sequence of the *eccB_5_-eccC_5_* locus in the used *M. tuberculosis* H37Rv strain. Sequence reads generated by next generation sequencing of *M. tuberculosis* H37Rv lined up below the previously reported *M. tuberculosis* H37Rv reference sequence. From this alignement the presence of a T instead of an A at position 2020563 is clearly visible.
(TIF)

Figure S3 Model of the Pip ON/Tet OFF repressible system in the Mtb*_Pptr_eccB_5_-eccC_5_* and Mtb*_Pptr_*eccC_5_ mutants. Schematic representation of the Pip ON/Tet OFF mycobacterial repressible circuit regulating the expression of *eccB_5_-eccC_5_* operon and *eccC_5_* gene in the Mtb*_Pptr_eccB_5_-eccC_5_ and* Mtb*_Pptr_*eccC_5_ conditional mutants, respectively, in the absence or in presence of ATc.
(TIF)

Table S1 Sequences of primers used in the study.
(PDF)
