# Supplementary figures and images for: The ESX-5 Associated eccB5-eccC5 Locus Is Essential for Mycobacterium tuberculosis Viability
Source: PLoS One. 2012 Dec 17;7(12):e52059. doi: 10.1371/journal.pone.0052059 (PMC3524121; doi:10.1371/journal.pone.0052059)

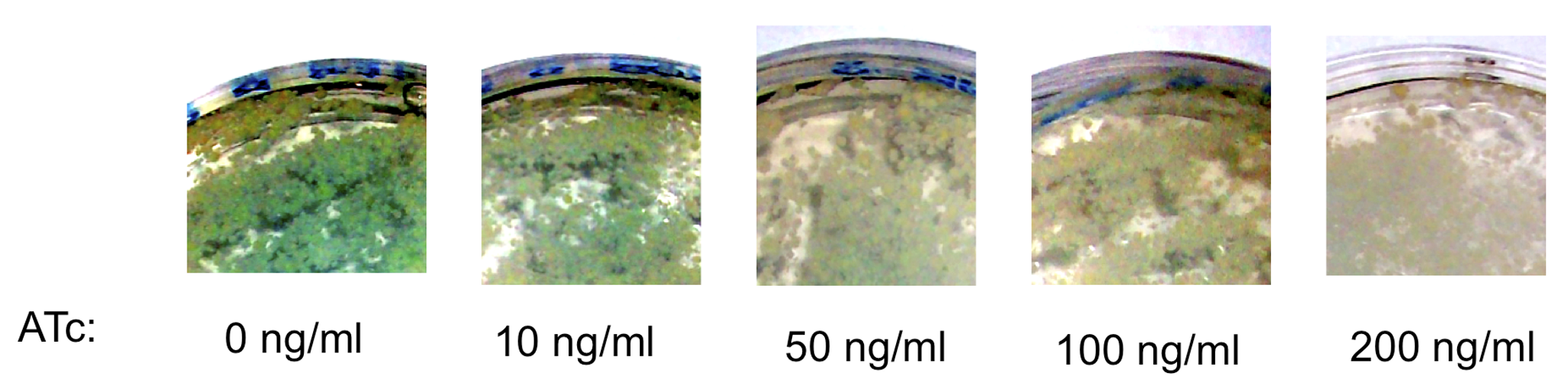

Supplement: Figure S1 — Characterization of the TetR/Pip OFF system in the Mtb::tetR-pip strain. ß-galactosidase assay performed on Mtb::tetR-pip strain grown on Middlebrook 7H11 plates containing X-gal (40 µg/ml) and different concentrations of ATc, ranging from 0 to 200 ng/ml. The ß-galactosidase activity was clearly detected when bacteria were grown in the absence of ATc, and decreased in the presence of increasing concentrations of the antibiotic. The ß-galactosidase activity was abolished when Mtb::tetR-pip was grown on Middlebrook 7H11 medium containing 200 ng/ml ATc. (TIF) [file pone.0052059.s001.tif]

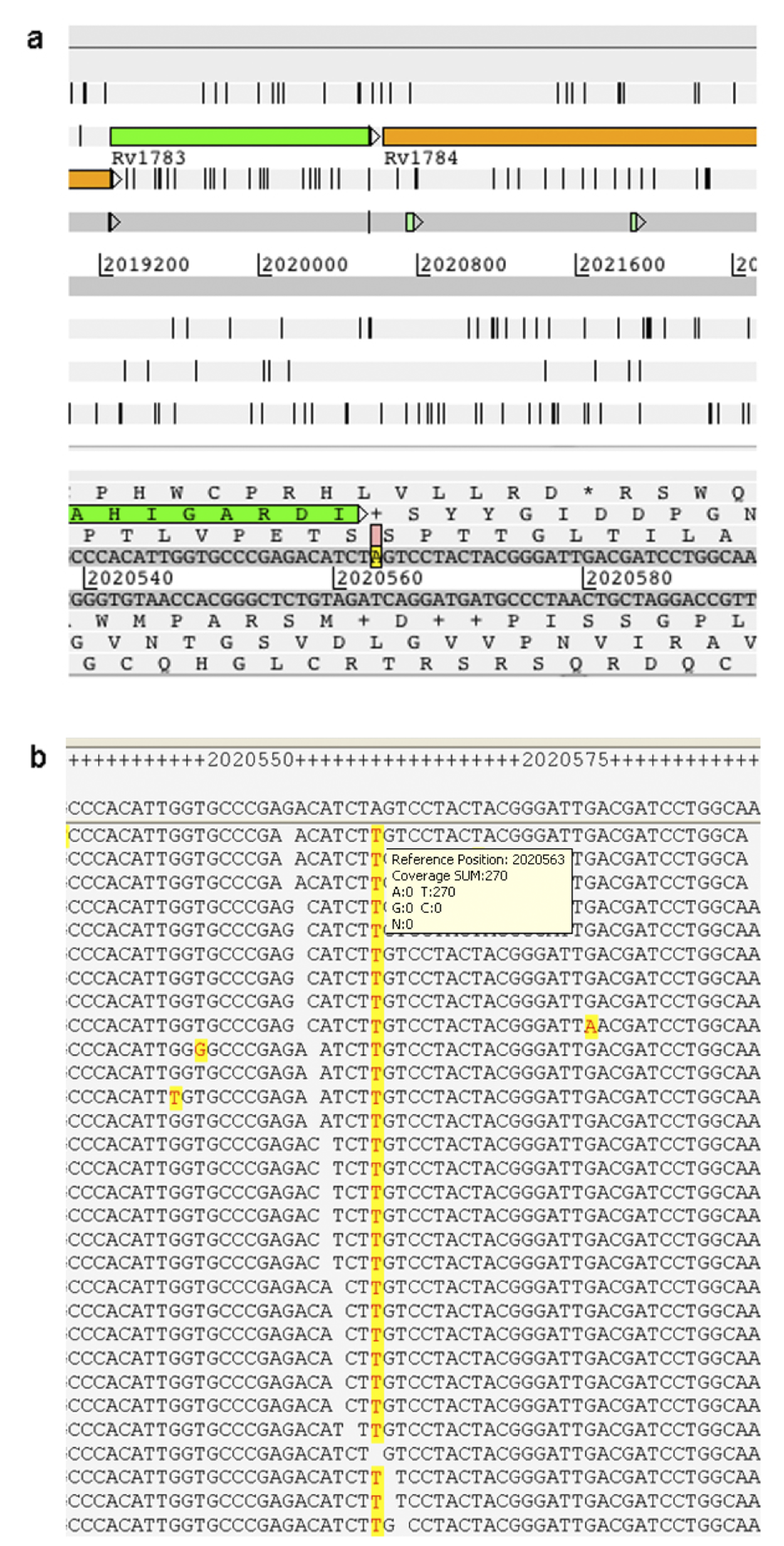

Supplement: Figure S2 — Sequence of the eccB5-eccC5 locus in the used M. tuberculosis H37Rv strain. Sequence reads generated by next generation sequencing of M. tuberculosis H37Rv lined up below the previously reported M. tuberculosis H37Rv reference sequence. From this alignement the presence of a T instead of an A at position 2020563 is clearly visible. (TIF) [file pone.0052059.s002.tif]

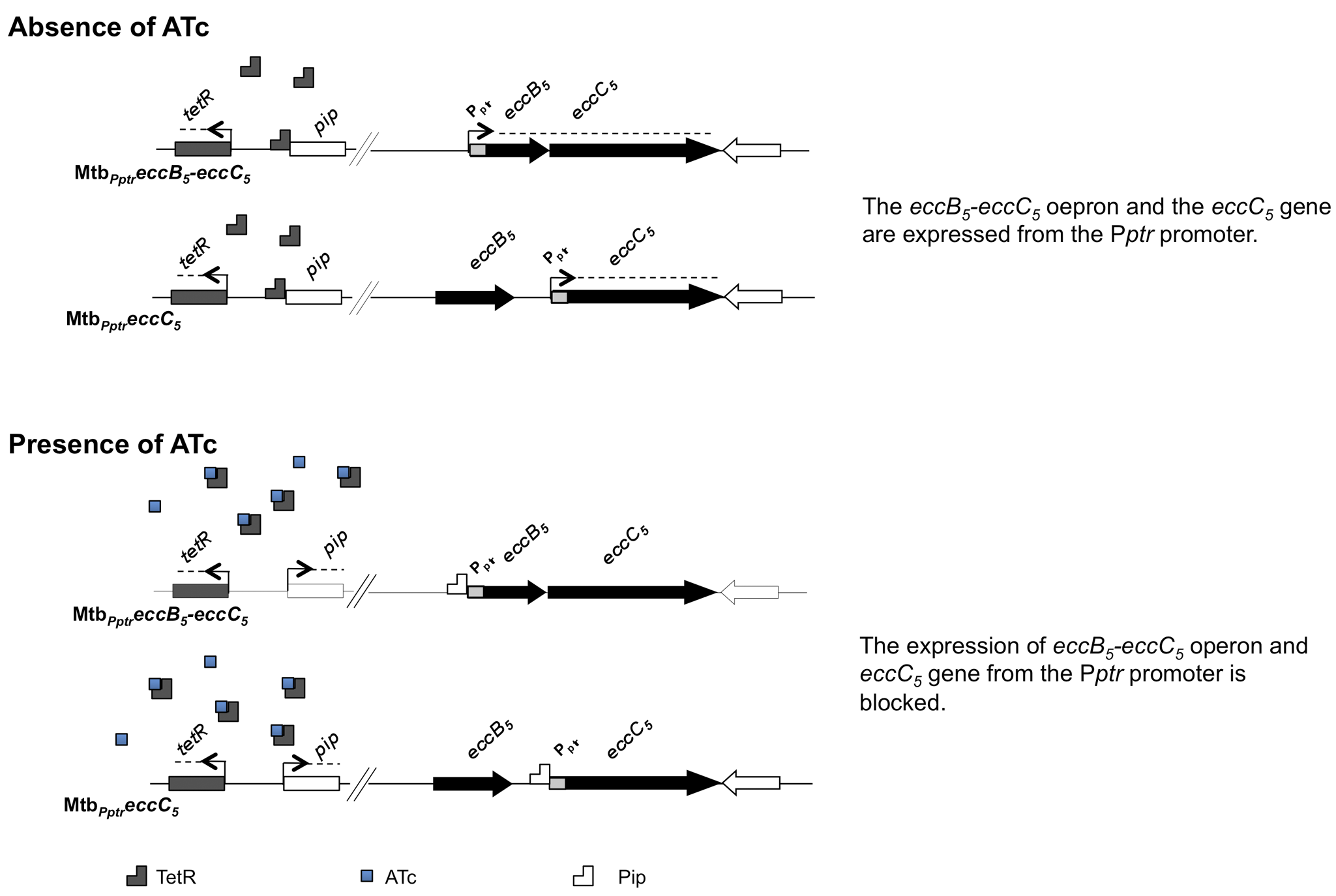

Supplement: Figure S3 — Model of the Pip ON/Tet OFF repressible system in the MtbPptreccB5-eccC5 and MtbPptreccC5 mutants. Schematic representation of the Pip ON/Tet OFF mycobacterial repressible circuit regulating the expression of eccB5-eccC5 operon and eccC5 gene in the MtbPptreccB5-eccC5 and MtbPptreccC5 conditional mutants, respectively, in the absence or in presence of ATc. (TIF) [file pone.0052059.s003.tif]
