# Supplementary material for: The ESX-5 Associated eccB5-eccC5 Locus Is Essential for Mycobacterium tuberculosis Viability
Source: PLoS One. 2012 Dec 17;7(12):e52059. doi: 10.1371/journal.pone.0052059 (PMC3524121; doi:10.1371/journal.pone.0052059)
Supplement: Table S1 — Sequences of primers used in the study. (PDF) [file pone.0052059.s004.pdf]

Table S1. Sequence of primer used in this study. Specific endonuclease restriction sites are indicated in bold.

**gene**	**Sequence (*restriction endonuclease*)**	**Application**
eccB$_5$ (*rv1782*)	AGATGAACAAGGTGGTGTCGT<br>ATTGGGAGCGTGTCGTGCTC	Expression studies, RT-PCR
eccC$_5$ (5' terminus) (*rv1783*)	ATCAAGCCCGAGAATATTGTCC<br>ATGTCCAGCATCAACATGAACT	Expression studies, RT-PCR
eccC$_5$ (3' terminus) (*rv1784*)	CTGCTGTTCTTGGATATGAAAT<br>TTCGAGGTCGGTGATGATCC	Expression studies, RT-PCR
eccB$_5$	TCCACTCAAAATCTGCCGGT<br>GACTCTTTGGCGAGAACGAT<br>ATCTGGCTTGACCGAACCAG	Retrotranscription in 5'RACE reactions<br>1st nested amplification in 5'RACE reactions<br>2nd nested amplification in 5'RACE reactions
eccC$_5$	CCAACCACAATCAGCCACCA<br>GTGTCGATAGGACAATATTCTC<br>ATGTTGAGGTAGGTGCCGAC	Retrotranscription in 5'RACE reactions<br>1st nested amplification in 5'RACE reactions<br>2nd nested amplification in 5'RACE reactions
eccB$_5$upstream region	GG**ACTAGT**ACCGAAAGCTCACCAACAG (*SpeI*)<br>GC**TCTAGA**TAGATCACCTTCCGGTCTGC (*XbaI*)	Amplification of *eccB*$_5$ upstream region for knockout construction
eccC$_5$ downstream region	GC**TCTAGA**TTCGGCCAAACCGATCAGCT (*XbaI*)<br>ATAAGAAT**GCGGCCGC**ACAAGAACGGAGACACGAAAG (*NotI*)	Amplification of *eccC*$_5$ downstream region for knockout construction
aph	GC**TCTAGA**GCTGCAAGGCGATTAAGT (*XbaI*)<br>GC**TCTAGA**ATTAGGCACCCCAGGCTTTA (*XbaI*)	Amplification of *aph* cassette
rv1780<br>*aph*<br>*rv1786*<br>*aph*	TCATCAATGTGGCCGAACAATT<br>GCAATGTAACATCAGAGATTTTGAG<br>ATAGCACTGCGCATGACCCA<br>CACGAGGCAGACCT	Screening of Kana^r/Sac^r transformants
eccB$_5$<br>eccC$_5$	GG**ACTAGT**GTGGCTGAAGAGAGCCGCGG (*SpeI*)<br>AATC**GTTAAC**CTACCGACGCACCTCGGTG (*HpaI*)	Construction of Mtb488 merodiploid strain
eccB$_5$	TGC**ATGCAT**GCTGAAGAGAGCCGCGGG (*NsiI*)<br>TGC**ATGCAT**ACTACCAGCGTTCTGCAG (*NsiI*)	Construction of Mtb$_{Pptr}$*eccB*$_5$-*eccC*$_5$ conditional mutant
eccC$_5$	TGC**ATGCAT**AAGCGTGGTTTTGCCCGCC (*NsiI*)<br>TGC**ATGCAT**CAAACTCACGAACCGAGGTG (*NsiI*)	Construction of Mtb$_{Pptr}$*eccC*$_5$ conditional mutant
ptr promoter<br>*eccB*$_5$<br>*eccC*$_5$	CAGCGTATGGGAATCTCTTG<br>AGAGAGATCCAACCGATTCAC<br>CGTGTCGAGGCGTGAAAGAA	Screening of Mtb$_{Pptr}$*eccB*$_5$-*eccC*$_5$ and Mtb$_{Pptr}$*eccC*$_5$ strains
